# Dark Field Microscopy-Based Biosensors for the Detection of *E. coli* in Environmental Water Samples

**DOI:** 10.3390/s19214652

**Published:** 2019-10-26

**Authors:** Rita La Spina, Diana C. António, Cloe Desmet, Andrea Valsesia, Radoslaw Bombera, Hedvig Norlén, Teresa Lettieri, Pascal Colpo

**Affiliations:** European Commission, Joint Research Centre (JRC), Via E. Fermi 2749, 21027 Ispra, Varese, Italydiana_conduto@hotmail.com (D.C.A.); Andrea.VALSESIA@ec.europa.eu (A.V.); hedvig.norlen@ec.europa.eu (H.N.); Teresa.LETTIERI@ec.europa.eu (T.L.)

**Keywords:** bacteria quantification, antibodies, dark field microscopy, field water samples

## Abstract

Development of sensitive methods for the determination of *E. coli* bacteria contamination in water distribution systems is of paramount importance to ensure the microbial safety of drinking water. This work presents a new sensing platform enabling the fast detection of bacteria in field samples by using specific antibodies as the biorecognition element and dark field microscopy as the detection technique. The development of the sensing platform was performed using non-pathogenic bacteria, with the *E. coli* DH5α strain as the target, and *Bacillus sp.* 9727 as the negative control. The identification of the captured bacteria was made by analyzing the dark field microscopy images and screening the detected objects by using object circularity and size parameters. Specificity tests revealed the low unspecific attachment of either *E. coli* over human serum albumin antibodies (negative control for antibody specificity) and of *Bacillus sp*. over *E. coli* antibodies. The system performance was tested using field samples, collected from a wastewater treatment plant, and compared with two quantification techniques (i.e., Colilert-18 test and quantitative polymerase chain reaction (qPCR)). The results showed comparable quantification capability. Nevertheless, the present method has the advantage of being faster, is easily adaptable to in-field analysis, and can potentially be extended to the detection of other bacterial strains.

## 1. Introduction

Microbial contaminations of drinking water represent a major issue in both developed and developing countries since it may cause a high level of infectious diseases and mortality [1]. Thus, the determination of *E. coli* bacteria resulting from the potential fecal contamination of water distribution systems is of paramount importance to ensure the microbial safety of drinking and bathing water reservois. High performance sensing devices are thus required to monitor water quality in the field. Current methods used for bacteria detection are based on culturing techniques, quantitative polymerase chain reaction (qPCR), and immunological reactions [2]. These methods are widely used and are very robust, but present some drawbacks. For instance, bacteria culturing techniques are time-consuming, requiring up to 24 h of incubation for cell quantification. Quantitative polymerase chain reaction (qPCR) offers highly sensitive and quantitative analyses, but requires expensive reagents and specific expertise. Similarly, the enzyme-linked immunosorbent assay (ELISA), which relies on specific antibodies for pathogen detection, requires a high level of automation due to multiple incubations and washing steps [3]. Modern biosensors are a promising alternative to detect bacteria in a rather short time and from a small amount of sample [4]. Biosensing devices can be developed by using antibodies immobilized on the sensor surfaces as bio-recognition elements. Label-free biosensors present many advantages such as a short time of the assay, but may have some limitations in terms of the detection specificity of the transduced signal. Indeed, non-specific attachment on the surface may result in some altered or false positive signals. To tackle this issue, sandwich assays with labelled detection antibodies are often used for bacteria identification [5]. 

Fluorescence microscopy is an outstanding technique for bacteria imaging and enumeration providing detection at single cell level. Flow cytometry also enables a reliable enumeration and differentiation of dead and alive cells in a rapid manner [6]. Nevertheless, both methods require sample-treatment *i.e*. bacteria staining and rather costly instrumentation with limited portability. 

Dark field (DF) microscopy is an interesting, easy, and inexpensive alternative visualization technique to image unstained specimens. This technique has been successfully used to image bacteria [7] and protozoa [8]. DF microscopes have the advantage to be rather simple and cost effective with the good potential of portability [9].

This work describes a label-free detection platform to enable the fast detection of bacteria within the field-derived samples. The method is based on an in-flow sensing platform with DF microscopy detection [10]. The recognition of the bacteria was performed using polyclonal antibodies. The immobilization of the antibodies on the surface was optimized by Surface Plasmon Resonance (SPR). The identification of bacteria is specifically directed against lipopolysaccharides and lipoproteins present in the outer layer of the *E. coli* cell membrane wall. This assay was performed by incubating *E. coli* bacteria on the anti-*E. coli* antibody modified surface for 75 min. After surface washing, the bacteria attached to the surface were screened by the statistical analysis of images recorded by DF. The identification of *E. coli* bacteria was carried out based on their area (i.e., relative size) and circularity (i.e., orientation) analysis. The reliability and robustness of the proposed assay was assessed by comparing its performance with conventional methods such as the Colilert-18 test and qPCR by using samples collected from a wastewater treatment plant. 

## 2. Materials and Method

### 2.1. Chemicals

Phosphate buffer saline (PBS) and ultra-pure water were purchased from Gibco Italia. Peptone, meat extract, tryptone, yeast extract, and Noble Agar were purchased from BD Diagnostics (Milan, Italy,. Ethanolamine, 16-mercaptohexadecanoic acid, 1-ethyl-3-(3-(dimethylamino)-propyl) carbodiimide (EDC), N-hydroxy succinimide (NHS), and bovine serum albumin (BSA) were purchased from Merck KGaA, (Darmstadt, Germany). Antibodies anti-Human Serum Albumin (Monoclonal anti-HSA-11 A6684) were purchased from Merck KGaA, (Darmstadt, Germany) and anti-*E. coli* (Polyclonal anti-*E. coli* ab13627) from Abcam (Cambridge, UK). Customized primers and a probe for qPCR-based detection of *E. coli* were purchased from Primm Srl. TaqMan Universal Master Mix with Rox and 96-well reaction plates were purchased from Invitrogen. Colilert-18 reagent, sample vessels, and Quanti-Tray/2000 were purchased from IDEXX. Amicon Ultra centrifugal filter units of 50 kDa and Whatman filter membranes of 0.22, 5, and 20 μm porosity were purchased from Sigma-Aldrich (Darmstadt, Germany.

### 2.2. Bacterial Cultures

Bacterial strain *E. coli* DH5α was purchased from the American Type Culture Collection and *Bacillus sp.* 9727 strains were purchased from the Leibniz Institute DSMZ-German Collection of Microorganisms and Cell Cultures. All cells were maintained in rich media and kept at −20 °C for long storage. *E. coli* strains were cultured in Luria-Bertani medium (LB) at 35 °C and *Bacillus sp.* was cultured in Alkaline Nutrient broth (AN) at 30 °C with agitation, according to the manufacturer’s recommendations. Agar plates were prepared with LB or AN media by adding 15 g L^−1^ Noble Agar. Plates were used to determine the cell abundance based on colony forming unit (CFU) enumeration. To avoid unspecific interaction with media components, freshly grown cells were washed in PBS before analysis. For the experiments, cultures grown overnight were washed three times by centrifugation for 2 min at 10,000 rpm and resuspended in PBS to the desired concentration. The cell concentration was confirmed by absorption and CFU enumeration. Experiments were performed with cell concentrations ranging from 10^4^ to 10^7^ cells Ml^−1^.

### 2.3. Surface Functionalization for Dark Field Measurement

For the dark field experiment, double-sided polished silicon wafers (from the Institute of Electronics Material Technology, Warszawa, Poland) were coated with gold thin film (150–200 nm) by the DC magnetron sputtering technique. A 5 nm titanium layer was used to improve the adhesion between the silicon and gold. The gold surfaces were first cleaned by pure oxygen plasma (100 mTorr with a power of 55 W and bias of 400 V) for 2 min, immediately followed by incubation overnight in a solution of 1.44 mg mL^−1^ 16-mercaptohexadecanoic acid (16-MHC, Sigma-Aldrich, (Darmstadt, Germany) in 100% ethanol to form a self-assembled monolayer (SAM) that presented the carboxylic acid functional group at the surface. The samples were rinsed with 70% ethanol and dried carefully. The 6-channel cartridge (Ibidi Germany, μ-slide VI 0.4, volume of the channel: 30 μL, growth area: 60 mm^2^) was then assembled and glued to the gold surface to carry out the DF analysis. The –COOH groups of the SAM were activated using a mixture of N-hydroxysuccinimide (NHS, 0.1 M in MilliQ water, Sigma Aldrich) and ethyl(dimethylaminopropyl) carbodiimide (EDC, 0.4 M in MilliQ water, Sigma Aldrich) for 10 min at room temperature. Polyclonal anti-*E. coli* antibodies were used for bacteria detection and anti-HSA antibodies were used as the negative control. The surfaces were then deactivated with ethanolamine for 10 min and subsequently blocked with bovine serum albumin for 30 min.

### 2.4. Surface Plasmon Resonance Experiments

Surface plasmon resonance (SPR) [11] experiments were performed using the Biacore T200 system (GE Healthcare, Milan, Italy). Both antibodies (anti-*E. coli* and anti-HSA) were immobilized onto a Biacore sensor chip C1 consisting of a gold surface coated by a thin carboxylated layer. The antibodies were immobilized by the standard amine coupling procedure. First, the –COOH groups were activated by 10 min injection (10 µL min^−1^) of mixture composed of 50 mM NHS (N-hydroxysuccinimide) and 200 mM EDC (ethyl(dimethylaminopropyl) carbodiimide) in MilliQ water. The antibodies were then flowed onto the surface by two successive injections (10 min each) into two different channels. Each injection was composed of 50 µg mL^−1^ of antibody in 10 mM sodium acetate buffer (pH 5.5). Antibody immobilization was followed by a 10 min injection of 1 M ethanolamine (pH 8.5) to deactivate any remaining reactive groups. An additional channel was activated with EDC/NHS and submitted to ethanolamine without antibody immobilization in order to serve as a reference during SPR data treatment. Typically, the method resulted in ca. 2200 RU (Resonance Unit, SPR specific signal) of the antibody immobilized at each flow channel of the sensor surface. Prior to incubation with bacteria, the sensor surface was additionally blocked with a 30 min injection of 1 mg mL^−1^ BSA and equilibrated in PBS solution. *E. coli* suspensions in PBS were introduced in subsequent injections at concentrations of 10^6^, 10^7^, 10^8^, and 10^9^ cells mL^−1^. Bacteria were injected during 30 min with flow maintained at 1 µL min^−1^ followed by 5 min PBS wash at 50 µL min^−1^.

### 2.5. Field Water Samples

To validate the sensing platform, two field water samples were tested (noted as A and B). Samples were collected from a wastewater treatment plant (WWTP) after reducing the organic load in suspension and before the last step of treatment (UV irradiation). WWTP samples have a high organic load, which may contribute to unspecific interactions within the evaluated methods. The choice of sample intended to challenge the systems and evaluate their robustness at the same time. The collected water sample was then passed through a series of filtering steps. For sample A, the water sample was passed through a 45 µm metallic sieve followed by passage through a 20 and 5 µm porous filter membrane. For sample B, the water sample was passed through a 45 µm metallic sieve followed by a passage through a 20 µm porous filter membrane.

### 2.6. Dark Field Microscopy Analysis 

Prior to dark field analysis, in order to concentrate the field samples, filtrated water was centrifugated using the Amicon Ultra-15 with a 50 kDa cut-off (Millipore). In detail, 45 mL of filtrated water was concentrated to 100 µL and resuspended in 400 µL PBS, obtaining a final 90-fold concentration. Concentrated samples were flowed over anti-*E. coli* antibody modified channels for 5 min at the rate of 50 µL min^−1^. Afterward, the water samples were left to incubate in static mode, in contact with the surface for 75 min, and then rinsed with PBS for 20 min at 50 µL min^−1^. In order to enumerate the attached bacterial particles (counts), DF images of the remained bacteria on the surface were captured and processed (imaging stacking, contrast enhancement) using RS ImageJ software. The same experiments were performed with known amounts of *E. coli* cultured bacteria in order to generate a calibration curve of the initial concentration of bacteria versus the count of bacteria immobilized on the surface per field of view. The calibration curve was then used to convert counts of bacteria to cell concentration (cells mL^−1^) in the field samples. 

### 2.7. Colilert-18 Test

Filtered samples were used for Colilert-18 analysis without pre-treatment (IDEXX Laboratories Italia S.r.l.). Serial dilutions were performed in filtered MilliQ water to find the optimal test concentration for quantification. Total coliforms and *E. coli* cells were quantified using Colilert-18 and Quanti-Tray/2000 (IDEXX), according to manufacturer’s instructions.

Briefly, 100 mL of sample was mixed with Colilert-18 reagent until complete dissolution of the powder and transferred to the Quanti-Tray. Bubbles were removed before sealing the tray. A negative control was performed using filtered MilliQ water. Samples were incubated up to 20 h at 35 °C and quantified using the statistical matrix to find the most probable number of cells per 100 mL of sample (MPN/100 mL), as supplied by the company. Colilert-18 can be used to target fecal contamination, at 44 °C, however, in order to compare it with the used antibodies and qPCR primers, we chose not to restring the analysis and therefore used 35 °C to screen for total coliforms.

### 2.8. Quantitative Real-Time Polymerase Chain Reaction Analysis

Filtered samples (after passage through a 45 μm metallic sieve, as described in the section on dark field microscopy analysis) were processed for DNA extraction. Aliquots of 200 mL were filtered through 0.22 μm porous filter membranes in order to retain the bacterial fraction. Filters were stored at −20 °C until processing. DNA extraction was performed using a DNeasy Blood & Tissue Kit (Qiagen), according to the manufacturer’s instructions. Previous cell disruption steps were performed in order to optimize the extraction yield. Filter samples were placed in 8 mL of 50 mM KH_2_PO_4_ at pH 7.5 overnight to facilitate cell detachment from the filter membrane. Successively, a mechanic disruption step using 15 min bath sonication at 60 °C was performed followed by chemical and enzymatic digestion, where cells were incubated with 0.7% lysozyme and 0.1% β-mercaptoethanol for 3 h at 30 °C with 180 rpm agitation. To determine the impact of unviable cells and free DNA in the total quantification, a second set of samples was extracted after performing a DNA digestion step. DNA digestion was performed, directly on the filters, by using 50 U of DNase I in 500 mL of reaction buffer (Invitrogen), incubated for 15 min at room temperature, with constant agitation. DNase I was inactivated before the cell disruption steps with the addition of 50 µL 25 mM EDTA and incubated for 10 min at 65 °C. Filters were then transferred to 8 mL 50 mM KH_2_PO_4_ at pH 7.5 and DNA extraction was carried out as previously explained. Extracted DNA was cleaned using the sodium acetate and ethanol precipitation method and concentrated in a total of 20 µL AE buffer. It was consecutively quantified using the Qubit dsDNA HS Assay Kit (Thermo Fisher Scientific, Milan, Italy). Quantitative real-time polymerase chain reaction (qPCR) was performed using custom made primers and probe targeting *E. coli* 16S rRNA gene. The reaction was performed using the TaqMan™ Universal PCR Master Mix (Applied Biosystems™, Thermo Fisher Scientific, Milan, Italy), 0.9 µM of each primer (*E. coli 16S Fw*—CCCTTACGACCAGGGCTACAC and *E. coli 16S Rv*—GCGAGGTCGCTTCTCTTTGT) and 0.2 µM of the probe (*E. coli 16S P* - [MGB]CGTGCTACAATGGCGCA[6 FAM]) in an ABI PRISM 7000 Sequence Detection System (Applied Biosystems). Bacteria absolute quantification by qPCR was calculated by assuming an average presence of seven copies of the 16S rRNA gene per *E. coli* cell [12].

## 3. Results and Discussion

**Figure 1:**
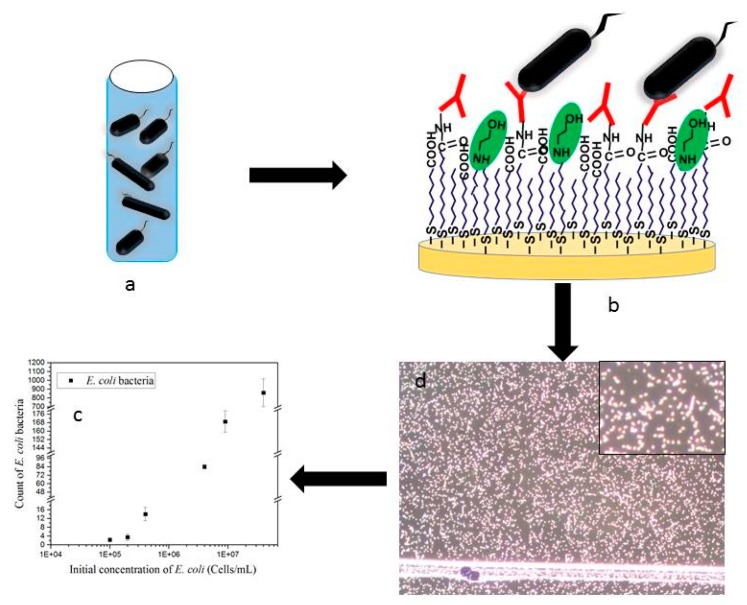
(**a**) Sample (spiked with *E. coli* or field sample) was flowed over (**b**) an anti-*E. coli* antibody functionalized gold surface. (**c**) Detection with dark field microscopy of a surface incubated with a field sample for 75 min and rinsed with phosphate buffer solution; enlargement of the picture. (**d**) Statistical image analysis was performed for the counting of the bacteria captured by antibodies.

The representation of the sensing platform is shown in Figure 1.

The different antibodies used in these experiments were immobilized on the gold coated surface using carbodiimide chemistry as described above. Each channel was then connected to a peristaltic pump to run the sample containing bacteria over the antibody-functionalized surface. The flow was stopped and the sample was incubated for 75 min on the sensing platform. The bacteria captured on the surface were then detected by dark field microscopy and statistical analysis was performed on the recorded image for the counting of the bacteria captured by antibodies.

### 3.1. Optimization of the Binding Assay in Surface Plasmon Resonance Measurements

**Figure 2:**
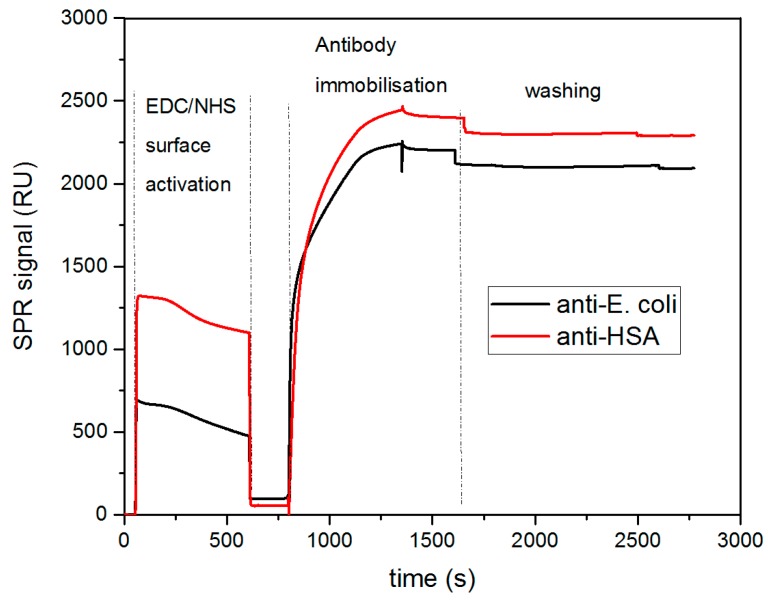
Real-time monitoring of anti-Human Serum Albumin and anti-*E. coli* immobilization by surface plasmon resonance. The gold surface was first activated with 1-ethyl-3-(3-(dimethylamino)-propyl) carbodiimide and N-hydroxy succinimide (EDC/NHS) chemistry and subsequently loaded with corresponding antibodies onto two separate channels. SPR curves were subtracted by a reference channel signal where no antibody was immobilized. Deactivation with ethanolamine and blocking operations with Bovine Serum Albumin are not shown.

SPR experiments were first carried out to characterize and optimize the immobilization of the antibodies on the gold surfaces as well as the capture of bacteria. In this study, the antibodies were covalently immobilized on the gold surface functionalized with a self-assembled monolayer (SAM) terminated with carboxyl functional groups. To enable antibody covalent coupling, the –COOH groups were activated using standard EDC/NHS activation chemistry. The carbodiimide activation (EDC) along with the addition of NHS created amine-reactive NHS-esters able to form stable amide bonds with primary amines exposed by antibody molecules (mainly Lysine moieties). SPR measurements confirmed the antibody immobilization, as shown in Figure 2. Two antibodies (anti-HSA and anti-*E. coli*) were immobilized onto two separate channels and the SPR signal was recorded, being referenced to the signal from a reference channel, which was not functionalized with any antibody. The first step of EDC/NHS activation was observed as expected with a small increase in the SPR signal and was followed by a considerable signal rise upon the injection of antibody solutions. In this step, the immobilization of anti-*E. coli* and anti-HSA antibodies is obtained, with an increase in signal of near 2300 RU and 2100 RU, respectively. Loading the antibodies at a concentration of 50 µg mL^−1^ ensured the stable immobilization of the bioreceptors, with a similar surface coverage observed for both antibodies. After deactivation and BSA blocking of the surface (data not shown), the biosensor was washed in PBS and the resulting SPR baseline remained stable for further measurements.

**Figure 3:**
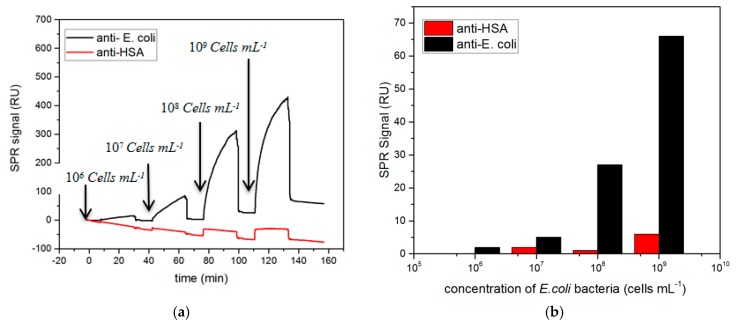
SPR evaluation of *E. coli* specific binding to anti-*E. coli* in comparison to anti-HSA (negative control). (**a**) SPR kinetic curves obtained during injections of *E. coli* from 10^6^ to 10^9^ cells mL^−1^. (**b**) SPR responses as a function of the concentration of bacteria (after washing in PBS. The SPR signal was obtained by subtracting the reference channel signal (without antibodies)).

Once the SPR biochips were functionalized with the antibodies, the detection specificity was assessed. The different channels were exposed to *E. coli* suspensions at increasing concentration. Figure 3a shows the SPR sensograms obtained during incubation of the bacteria onto anti-*E. coli* and anti-has antibodies functionalized surfaces. Figure 3b compares the SPR signal for each bacteria concentration, registered after each PBS washing step. The observed SPR curves demonstrated specific recognition of *E. coli* by the corresponding antibodies, with a signal increase proportional to the concentration of bacteria loaded on the channel. The response from the anti-HSA functionalized channel was found to be substantially lower when compared to the specific antibody. Interestingly, we observed a signal drop at the end of each injection, which was probably due to shear forces exercised on bacteria while washing in PBS. Nevertheless, the SPR signal remained still much higher for anti-*E. coli* than for the negative control (anti-HSA) as can be seen in Figure 3b. The described signal drop was certainly due to the particular behavior of bacterial cells in the confinement of SPR instrument microfluidic system. Indeed, the dimensions of *E.coli* were comparable to the tiny diameter of microfluidic channels available in Biacore T200 system. The bacteria, which are around 2 µm of diameter, were therefore subjected to shear stress, especially upon modification of the flow conditions (PBS washing after injection), leading to their desorption from the surface. Furthermore, the SPR responses to bacteria might not appear proportional but it is related to method limitation. SPR measurement enables the detection of changes in the refractive indexes at a distance of up to 150 nm from the sensing surface while the size of bacteria largely exceeds this scale, so the SPR signal results principally from the lower part of the bacterial wall in contact with the sensing surface. Moreover, the limited penetration of bacteria by the electromagnetic field and the similarity in refractive index between the bacterial cytoplasm and the aqueous medium is a drawback for the sensitivity of SPR in this application [13]. As such, SPR is probably not an optimal method for bacteria quantification. Nevertheless, the described SPR tests confirmed the correct immobilization of the antibodies onto the biochip surface and the subsequent specific recognition of *E. coli* bacteria. The SPR experimental conditions for the antibody immobilization were applied for the development of the biosensing platform using in DF detection methods.

### 3.2. Optimization of Experimental Conditions

The physical characteristics of the bacteria are an important parameter to consider for the development of the bioassay. Indeed, since the bacteria present large sizes and a mass density similar to water, the drag force due to the liquid flow tends to limit their transport to the sensing surface because of the low transport rate by diffusion and gravitational sedimentation. To address this issue, the assay has to be preferentially carried out in static mode. A minimum number of five bacteria immobilized on the surface was considered to have accurate counting within the used field of view (400 × 400 μm^2^). For this given field of view and incubation time of 75 min, 17 ± 3 bacteria were counted for a concentration of 4 × 10^5^ cells mL^−1^. No significant increase in the number of bacteria bound on the surface was observed for a longer exposure period. The duration of the experiments could be drastically decreased by increasing the counting area. For instance, for 15 min, five bacteria detected at 400 × 400 μm^2^ could be increased to more than 20 bacteria by increasing the sampling area by a factor of 4.

### 3.3. Dark Field Data Treatment

The detection of bacteria immobilized on the surface of the sensor was performed by dark field microscopy. Images were recorded after 75 min of sample incubation and 30 min of rinsing to remove loosely bound bacteria. ImageJ software was used to analyze the images and count the bound bacteria.

**Figure 4:**
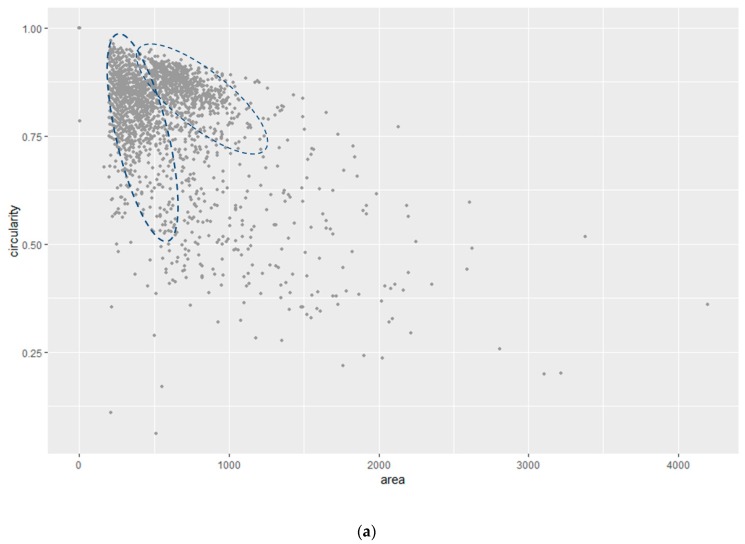

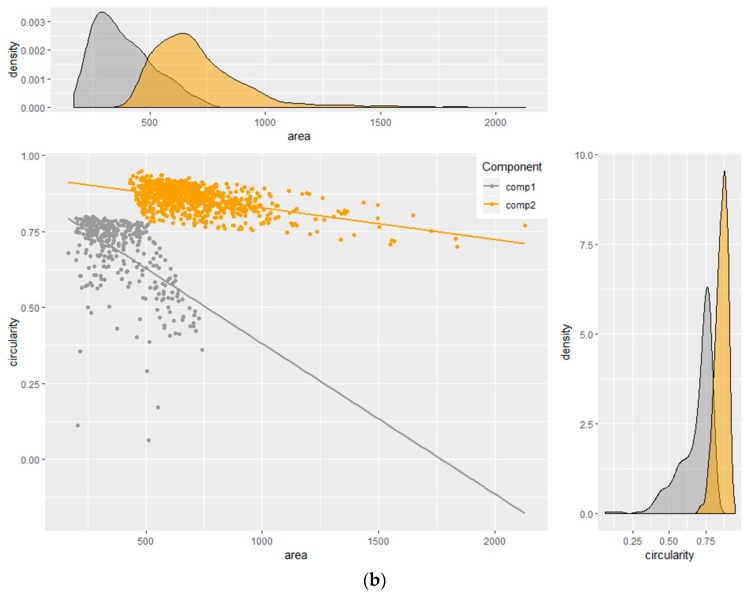
(**a**) Scatter plot of area (pixel^2^) versus the circularity measurements of *E coli* bacteria. (**b**) Mixture of regression models of the area (pixel^2^) versus circularity.

The average area and circularity distribution of the *E. coli* bacteria was determined by using all bacteria detected by ImageJ without geometric restrictions (Figure 1c is an example of the original microscopic image). These characteristics were further used to screen and quantify the amount of *E. coli* bacteria in a field sample. Figure 4 illustrates the measurements of area versus the circularity of a total sample of n= 2459 *E. coli* bacteria obtained from five independent experiments. The bacteria measurements were analyzed using mixtures of regressions models, which then enabled us to discriminate the shape and consider its binding orientation to the functionalized surface. Mixture regression models are applied when there are several mixed groups in the population that the sample represents. In this case, the *E. coli* population on the surface could assume several shapes versus orientation such as a round-shape and rod like shape. Round shapes can be observed when single bacteria assume an up-right position or several bacteria are associated together. In both scenarios, a circularity value close to 1 was measured. Furthermore, rod like shapes can be observed if a single bacteria is side-on to the surface (circularity lower than 1) or assume any intermediate position (circularity between 0 and 1). By modeling the conditional distribution of the response given for the covariate as a mixture, the sample can be clustered into groups and the regression models for the groups can be estimated at the same time. Mixtures of linear regression models were first studied in the econometrics literature and were introduced by Quandt [14] and Quandt and Ramsey [15] as the “switching regression” problem. There are several applications of mixture distributions in agriculture, biology, economics, medicine, and genetics (see e.g., [16] and [17] for reviews).

Figure 4a illustrates the presence of two, although not well separated, clusters in the sample. In the first instance, the measurements of circularity were modeled as the response of the area covariates. Suppose that we have *n* independent observations of circularity, $y_{1},\ldots ,y_{n}$, each with a corresponding predictor measurement of area, $x_{1},\ldots ,x_{n}$. Furthermore, each observation ($y_{i},x_{i})$ belongs to either one of two main groups of *E. coli* bacteria distribution. Conditional on the membership in the *j*th group (component), the relationship between $y_{i}$ and $x_{i}$ is the normal regression model and the mixture of regression models is given by:

(1) $$y_{i}=\{\begin{array}{c} \alpha_{1}+\beta_{1}x_{i}+\epsilon_{1i}\;\mathrm{with}\;\mathrm{probability}\;\lambda \\ \alpha_{2}+\beta_{2}x_{i}+\epsilon_{2i}\;\mathrm{with}\;\mathrm{probability}\;1-\lambda \end{array}$$

where the $\epsilon_{ji}\sim \;N\left(0,\sigma_{j}^{2}\right)$ are independent, j= 1, 2, i= 1, …, *n*.

The problem is to estimate the seven parameters that specify the proportion of points to each cluster (belonging to each regression line), the slope and the intercept of each line, and the variance around each line. The parameter vector is denoted as $\theta =\left(\lambda ,\alpha_{1},\alpha_{2},\beta_{1},\beta_{2},\sigma_{1}^{2},\sigma_{2}^{2}\right)$ and accounting for the mixture structure, conditional on $x_{i}$, $y_{i}$ has the density

(2) $$f_{\theta }(y_{i}|x_{i})=\lambda f_{1}(y_{i}|x_{i})+(1-\lambda )f_{2}(y_{i}|x_{i})$$

where $f_{j}(y_{i}|x_{i})$ is the normal density with a mean of $\alpha_{j}+\beta_{j}x_{i}$ and variance of $\sigma_{j}^{2}$, j= 1, 2. We assume that $x_{i},1\leq i\leq n$ are such that $\underset{n\to \infty }{lim}X^{\prime }X/n$ is positive definite. The expectation maximization (EM) algorithm, originally introduced by Dempster, Laird, and Rubin [18], has been used to compute the maximum likelihood estimators (MLE). The EM estimates are iteratively reweighted MLE with weights equal to the current conditional probability of the component membership. Second, the measurements of the area were modeled as the response of circularity with a mixture of regression models. The two mixtures of regression models were subsequently matched (i.e., when the calculated posterior probabilities of belonging to one component coincide). Through the matching, the clusters were further separated into two main components, which represented the average area and the circularity of *E. coli* bacteria. Specifically, the clusters could be separated in Component 1 and Component 2, and the estimated parameter vector was $\hat{\theta }$ = (0.17, 0.56, 0.78, 0.00012, 0.00010, 0.14^2^, 0.05^2^). The results of the average area ±σ and average circularity ±σ of *E. coli* bacteria are listed in Table 1 and represented in Figure 4. Data analysis was carried out in the R environment for statistical computing and visualization [19] using the following libraries: dplyr [20], mixtools [21], and mixture [22].

Bacteria immobilized on the surface can be divided in two populations as a function of their arrangement and orientation. The first population, represented by Component 2, is characterized by a circularity value close to 1 (i.e., 0.86) and the largest area, representing most objects grouping two or more bacteria. The second population (Component 1), characterized by a lower circularity (<1) and an area lower than Component 2, might indicate single *E. coli* bacteria with side-on or intermediate orientations onto the functionalized surface. Therefore, Component 1 seems to statistically represent the average area and circularity of single *E. coli* bacteria. In further experiments, Component 2 was not used for the statistical analysis of *E. coli* in the field water sample because of the lack of specific information regarding the average area and circularity of single bacteria.

In order to assess the reliability of the described counting method, Component 1 values were applied and compared to manual counting using an antibody functionalized surface exposed to a known concentration of cultured *E. coli* bacteria. The analysis of known samples suggests that using Component 1 values with one standard deviation (area 386 ± 127 and circularity 0.68 ± 0.12) is very restrictive. Indeed, for a field of view of 400 × 400 μm^2^ and incubation time of 75 min, 259 bacteria were manually counted for a known concentration of 4 × 10^6^ cells mL^−1^. The application of the statistical parameters (area 386 ± 127 and circularity 0.68 ± 0.12) generated 44 counts of bacteria, which represented only 17% of the total count of bacteria. By using Component 1 values with 1.5 and 2 standard deviations, the number of detected bacteria increased to 74 (28% of the total count of bacteria) and 214 bacteria (82% of the total count of bacteria), respectively. However, the 2σ results overlapped between Components 1 and 2 (Figure 4), so were not retained as suitable for counting single bacteria population. Therefore, the counting of bacteria on the functionalized surface was carried out by considering 1.5σ as the statistical error.

### 3.4. Biosensor Specificity Testing

**Figure 5:**
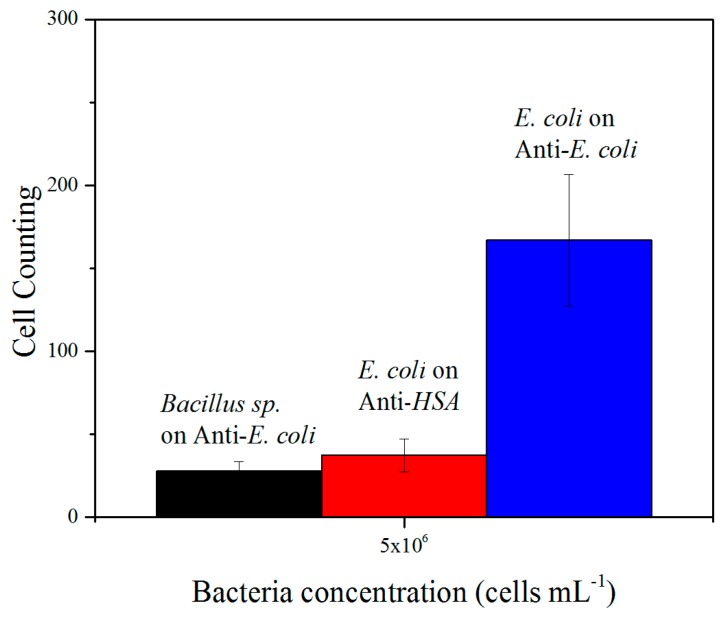
Comparison of bacteria binding, in counts, to different antibodies at a concentration of 10^6^ cells mL^−1^. *E. coli* bacteria were tested against anti-*E. coli* (positive control for specificity) and anti-HSA (negative control for specificity). A second negative control was performed using *Bacillus sp.* bacteria against anti-*E. coli*.

The specificity of detection was assessed by using surfaces functionalized with non-specific antibodies (anti-HSA) and bacteria non-specific to immobilized anti-*E. coli* antibodies (*Bacillus sp.* 9727 bacteria). The results presented in Figure 5 confirm those obtained with the SPR experiments (i.e., show low unspecific bacteria binding onto the anti-HSA functionalized surfaces). Similarly, the binding of *Bacillus sp.* 9727 bacteria onto anti-*E. coli* functionalized surfaces was limited (Figure 5), confirming the specificity of the used antibody and of the functionalized surface.

### 3.5. Biosensor Calibration

After optimization of the assay, the detection limit of the system was determined by spiking cultured *E. coli* bacteria in PBS solution. The flow cell was filled with 30 μL of PBS solution with an increasing concentration of bacteria (from 10^4^ to 10^7^ cells mL^−1^). The experiments were performed in triplicate. The bacteria solutions were flowed over the sensor surface and left to sediment toward the surface for 75 min. The surface was then washed with PBS for 20 min and the number of bacteria immobilized on the surface was counted by ImageJ after DF image recording. The field of view used for the counting was 400 × 400 μm^2^.

**Figure 6:**
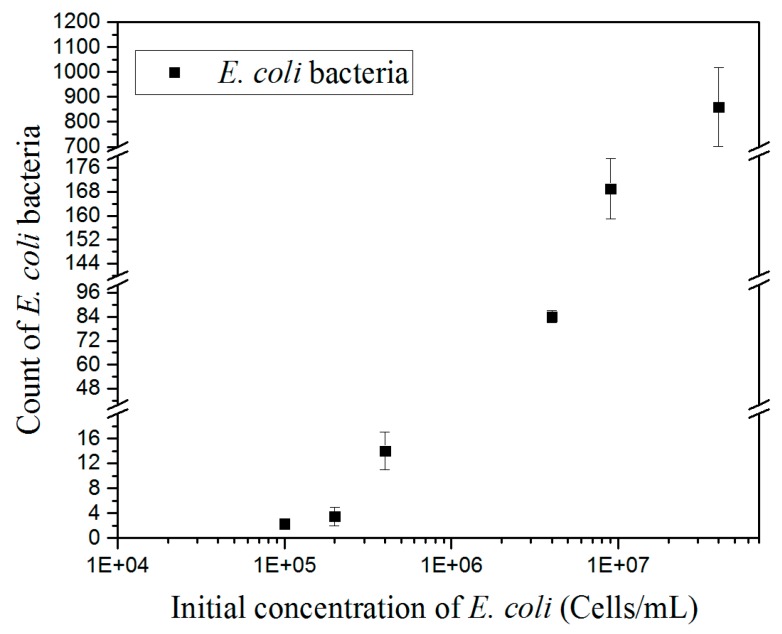
Calibration curve of the count of *E. coli* bacteria immobilized on the surface after the washing step versus the concentration of cultured *E. coli* bacteria.

The counting results of immobilized bacteria at different concentrations are shown in Figure 6. Increasing concentrations of *E. coli* generated a proportional increase of the number of bacteria immobilized on the surface. Quantitative detection of bacteria was carried out by considering a threshold of at least five bacteria for ensuring reliable counting [7], which is defined here as the detection limit. This reached a concentration of 2.10^5^ cells mL^−1^. Initial concentrations of *E. coli* bacteria of 4 × 10^5^, 4 × 10^6^, and 4 × 10^7^ cells mL^−1^ generated a number of immobilized *E. coli* bacteria of 15, 80, and 800, respectively, within an error of 4% and 18% for the highest concentration. The calibration curve could be fitted with a linear regression with R^2^ of 0.9. Nevertheless, increasing the DF sampling area from 0.16 mm^2^ to 1 mm^2^ would improve the quantitative detection of bacteria by a factor of 5–6 and reach a detection limit in the order of 5 × 10^4^ cells mL^−1^, which are values comparable to other conventional methods.

### 3.6. Application to Field Samples and Comparison of Techniques

**Figure 7:**
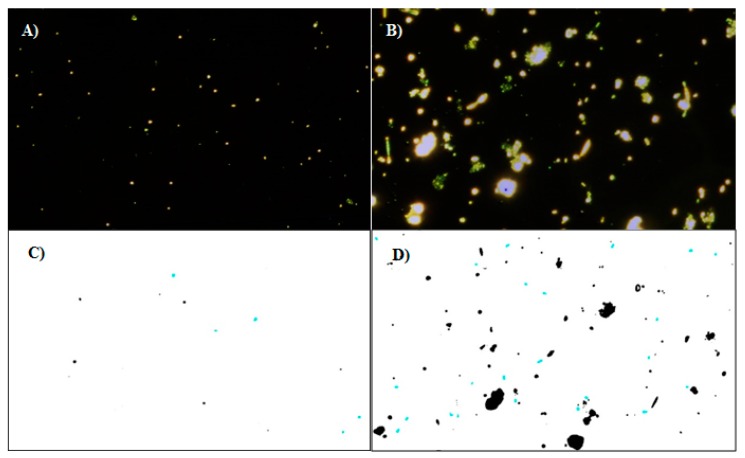
Bioassay performed on the field water sample filtered with a membrane with (**A**) 5 µm pores and (**B**) 20 µm pores. (**C**,**D**) Visual demonstration of bacteria screening using shape and area factors. The images corresponding to the set parameters appear in blue. The image represents the DF data obtained with a field sample and treated using ImageJ software.

The presently developed method was compared to two conventional methods, namely Colilert-18 and qPCR. The Colilert-18 method is an ISO standard (ISO 9308-2:2012) for the detection of total coliforms and *E. coli*. It has been demonstrated that the results are equivalent to traditional quantification methods [23] and it is included in the Drinking Water Directive [24]. The Colilert-18 method is very simple and does not require specific expertise and the results are obtained within 18 to 22 h. Quantitative PCR is another technique that is widely used for the quantification of known organisms. This technique has the potential to detect very low copy numbers of target genes. For quantification, a specific primer set is needed, which solely detects the region of interest of the target gene. Quantification may be done based on a non-specific dsDNA intercalating dye (SYBR Green) or by using a probe containing reporter and quencher molecules. The fact that different species, and even strains, may contain a different number of copies of a single gene impacts the extrapolation of the cell number based on qPCR analysis (which reports data as a gene copy number). For instance, it is of general agreement that *E. coli* has a total of seven copies of the 16S rRNA gene, and therefore the cell quantification must take that into account. Moreover, the maintenance of extracellular DNA in field samples is a fact and may impact the quantification of the total cell number if it is not taken into consideration. Pre-digestion of extracellular DNA before cell disruption and successively intracellular-DNA extraction reduces the impact of non-viable cells/DNA on the quantification analysis. Other methods can be used to this end such as the relative quantification of the extracted RNA, however, this would increase the length of the analysis. The DNA extraction process may vary depending on the protocol used. Nevertheless, the correlation between qPCR and culturing methods for the detection of coliforms and *E. coli* is known [25]. In order to assess the robustness of the DF based bioassay, experiments were performed by using two water samples from the wastewater treatment plant (Samples A and B), which were subsequently filtered through a 45 µm metallic sieve and 5 µm (Sample A) and 20 µm (Sample B) filter membranes. In the field water sample, the expected level of *E. coli* was estimated in the order of 10^4^ cells mL^−1^. Since our assay works in the order of 10^5^ cells mL^−1^ (i.e., the range of the calibration curve was from 5 × 10^4^ to 5 × 10^7^ cells mL^−1^), the field water samples had to be concentrated. DF images of the bacteria bound to the surface were captured and processed using ImageJ software (Figure 7). The screening of *E. coli* bacteria was performed by using the Component 1 parameters with ±1.5σ. These parameters were previously discussed in the dark field data analysis treatment paragraph. The count of *E. coli* bacteria was then converted in cells mL^−1^ by using the calibration curve seen in Figure 6.

### 3.7. Comparison of Techniques

**Figure 8:**
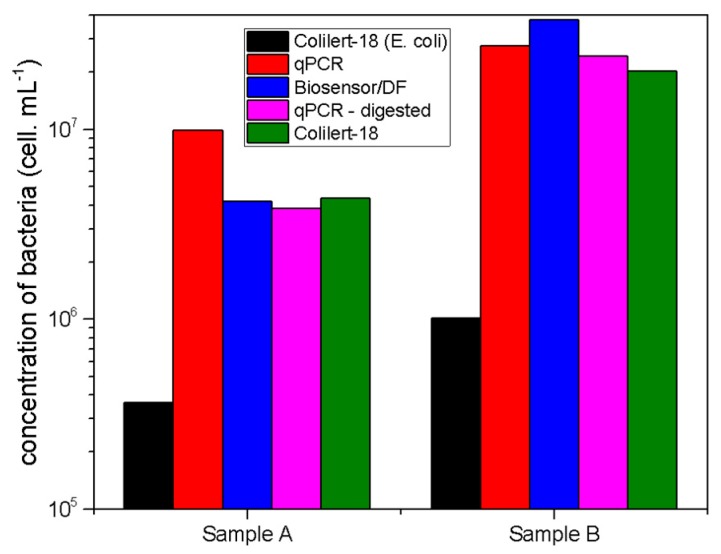
Plot comparing *E. coli* quantification based on the three tested methods: Colilert-18, qPCR, and biosensor-dark field. Quantification of total coliforms by the Colilert test (in green) was added as a reference. Quantification by qPCR is presented for both the pre-digested and raw samples.

Table 2 and Figure 8 show the results obtained by the different methods and the method variations used in this work.

Overall, the results were consistent and comparable among the techniques. The results obtained specifically for *E. coli* by the Colilert-18 method exhibited the lowest quantification in both samples when compared to the other methods. This effect can be explained by qPCR and biosensor technique pitfalls in term of specificity. Indeed, the results obtained with these methods were close to the ones obtained with the Colilert-18 method for the total coliforms by relying on the monitoring of a color-based chemical transformation (i.e., the metabolic conversion of o-nitrophenyl into o-nitrophenol by the β-galactosidase enzyme), which detected the presence of all viable cells in the sample. We could then assume that the results of all techniques considered not only the *E. coli* strain detected by Colilert-18, but also other coliforms. The results of the Colilert-18 tests were considered as a reference since it is the most accepted standard.

Over-estimation observed from the DF measurements, in addition to the other coliforms, could be attributed to the detection of non-viable cells (which will not grow in Colilert-18 media, and therefore could not be detected). Furthermore, the attachment may also be partially impaired by the organic load present in wastewater samples, which may affect the specific interaction with the antibody.

Similarly, qPCR analysis also showed an over-estimation due to a lack of specificity. To investigate the reason, we used BLASTN 2.8.0+ software [26] (NCBI) to analyze the qPCR target sequence CCCTTACGACCAGGGCTACACA*CGTGCTACAATGGCGCA*TACAAAGAGAAGCGACCTCGC, which was found by overlapping the primers/probe pair (primers underlined and probe in italic) with the complete genome of *E. coli* O157:H7 str. Sakai DNA (NCBI code >gi|47118301 |dbj|BA000007.2). BLAST index results [27] showed a high similarity with other bacteria species (even full identity) (data not shown). The lack of specificity of the primer set used in this study may explain the over-estimation of *E. coli* by qPCR in Sample B and the non-digested samples. On the other hand, the contaminants expected on a DNA extract of the WWTP samples may interfere with the PCR reaction. Inhibition of the enzyme was expected, however, the increase may somehow be an impact of the contaminants, which was not further evaluated.

The observed over-quantification was roughly 10 times higher for qPCR and the biosensor when compared to the Colilert-18 test for *E. coli*. Considering the Colilert-18 test results for coliforms, all methods performed similarly, which supports the theory that the proposed method may unspecifically recognize other coliforms due to low specificity of the polyclonal antibodies, unspecific recognition due to the presence of organic compounds or other contaminants, or other interactions that were not explored.

## 4. Conclusions

The sensing platform presented in this paper enables the specific detection and quantification of bacteria in complex samples by using dark field microscopy. The cell counting was performed with the average size and circularity criteria of the model bacteria strain used to plot the standard curve, which was the number of immobilized bacteria onto the surface vs. the initial concentration of bacteria. Quantitative detection of bacteria was carried out by considering a threshold of at least five bacteria in 0.160 mm^2^ to ensure reliable counting, and reached a detection limit of 2 × 10^5^ cells mL^−1^, which could be lowered to the order of 5 × 10^4^ cells mL^−1^ simply by increasing the DF sampling area. In addition, the platform and the methods showed high specificity toward the detection of coliforms. Despite the restrictions of the proposed detection method regarding specificity, it enables the quantification of bacteria-equivalents, even in field samples. The Colilert-18, qPCR, and DF quantification data were comparable in the detection of coliforms, thus proving the potential of the proposed method. Overall, the method presents a solution for reducing the water sample analysis time from 18 h for Colilert-18, to near 2 h using the biosensor coupled to DF microscopy. Another potential advantage of the proposed method is the possibility of transposing it to a portable system for in-field analysis.

## Figures and Tables

**Table 1:** Statistical values of the two components. Average area ±σ_1_ and circularity ±σ_2_ of *E. coli* bacteria by using the mixture of regression models.

Components	Area (pixels^2^)	*σ*_1_	Circularity	*σ*_2_
Component 1	386	127	0.68	0.12
Component 2	710	211	0.86	0.04

**Table 2:** Quantification of bacteria, in cells L^−1^, in Samples A and B by the IDEXX (Colilert-18), dark field (DF), and quantitative polymerase chain reaction (qPCR) methods.

Water Sample	Colilert-18 (*E. coli*, cells L^−1^)	Colilert-18 (Coliform, cells L^−1^)	Cell counting by DF (FoV 400 × 400 µm^2^)	Corresponding Cell Concentrations by DF (*E. coli*, cells L^−1^)	qPCR (Copy number L^−1^)	qPCR (*E. coli*, cells L^−1^)
A	3.6 × 10^5^	4.35 ×10^6^	8	3.25 × 10^6^	6.9 × 10^7^	9.91 × 10^6^
B	1.01 × 10^6^	2.02 × 10^7^	66	3.9 × 10^7^	1.92 × 10^8^	2.75 × 10^7^
A^a^	n/a	n/a	n/a	n/a	2.68 × 10^7^	3.83 × 10^6^
B^a^	n/a	n/a	n/a	n/a	1.71 × 10^8^	2.44 × 10^7^

^a^ DNA digestion of free material before extraction. n/a, not applicable.
